# Anti-inflammatory and anti-fibrotic effects of berberine-loaded liquid crystalline nanoparticles

**DOI:** 10.17179/excli2023-6467

**Published:** 2023-10-26

**Authors:** Amlan Chakraborty, Keshav Raj Paudel, Chao Wang, Gabriele De Rubis, Dinesh Kumar Chellappan, Philip Michael Hansbro, Chrishan S. Samuel, Kamal Dua

**Affiliations:** 1 Faculty of Biology, Medicine and Health, The University of Manchester, Oxford Road, Manchester M13 9PL, U.K.; 2 Cardiovascular Disease Program, Biomedicine Discovery Institute and Department of Pharmacology, Monash University, Clayton, VIC 3800, Australia; 3 Centre for Inflammation, Centenary Institute & University of Technology Sydney, School of Life Sciences, Faculty of Science, Sydney, New South Wales 2050 & 2007, Australia; 4 Discipline of Pharmacy, Graduate School of Health, University of Technology Sydney, Ultimo, NSW 2007, Australia; 5 Faculty of Health, Australian Research Centre in Complementary and Integrative Medicine, University of Technology Sydney, Ultimo, NSW 2007, Australia; 6 Department of Life Sciences, School of Pharmacy, International Medical University (IMU), Bukit Jalil, Kuala Lumpur, 57000, Malaysia

## ⁯

Inflammation is a common protective response activated within several tissues and organs upon exposure to potentially damaging or threatening stimuli. When the inflammatory response fails to neutralize the initiating trigger, inflammation persists for a prolonged time until reaching a harmful new homeostatic state termed chronic inflammation. In the respiratory tract, chronic inflammation is the main pathophysiological feature underlying chronic respiratory disorders (CRDs) such as asthma, chronic obstructive pulmonary disease (COPD), and pulmonary fibrosis (Clarence et al., 2022[4]). One of the most common airborne inflammatory triggers, which is known to initiate inflammatory cascades in the lungs, is the bacterial lipopolysaccharide (LPS), a fundamental component of the outer membrane of Gram-negative bacteria. LPS activates inflammatory responses by binding to the Toll-like receptor 4 (TLR4) expressed by tissue-resident macrophages and dendritic cells. This activates a complex network of signaling pathways resulting in the polarization of macrophages towards the M1 pro-inflammatory phenotype, with concomitant overexpression of the activation marker CD40 (Vogel et al., 2014[12]). 

In CRDs, persistent inflammation results in tissue damage and airway remodeling, a process consisting of a series of irreversible structural and functional alterations of the airways and lung parenchyma that is initiated upon tissue damage by the hormone transforming growth factor-β1 (TGF-β1) and includes subepithelial fibrosis, epithelial-to-mesenchymal transition, and aberrant collagen deposition (Rosethorne and Charlton, 2018[10]). 

The current treatments available for CRDs only target acute exacerbations and have limited efficacy in addressing underlying mechanisms of disease such as inflammation and remodeling. In this context, herbal medicine is gaining prominence as a valid alternative to conventional therapies. Berberine is an alkaloid molecule widely used in Chinese Traditional Medicine and extracted from barberry plants which is renowned for its wide range of antioxidant, anti-inflammatory, and antifibrotic properties. However, its clinical use is limited by poor permeability (Tew et al., 2020[11]; Paudel et al., 2022[8]).

In this study, we overcame the problem of permeability by encapsulating berberine in monoolein-based liquid crystalline nanoparticles (BM-LCNs) and investigated their (i) anti-inflammatory potential utilizing LPS-primed mouse-derived bone marrow derived dendritic cells and macrophages, as well as their (ii) anti-fibrotic potential by stimulating human dermal fibroblast with TGF-β1 to promote myofibroblast differentiation.

BM-LCNs were prepared by probe sonication method as described previously (Paudel et al., 2022[8]). To investigate the anti-inflammatory effects of BM-LCNs, we utilized murine bone marrow-derived dendritic cells and macrophages (BMDCs, CD11c^hi^,(Chakraborty et al., 2018[2])) characterized as CD11c^+^MHCII^+^Lin^-^ cells and mice bone marrow-derived macrophages (BMDMs, CD11c^neg^CD45^pos^ cells, (Deng et al., 2013[5]; Palmieri et al., 2020[7])) characterized as CD11c^-^MHCII^+^CD45^+^Lin^-^F4/80^+^ cells. The cells were treated with LPS (100 ng/ml for 24 hours) to induce activation and priming of the BMDCs and BMDMs. Following LPS treatment, we exposed the cells to BM-LCNs (100 μl of formulation with 1 ml formulation containing 200 mg Monoolein, 20 mg Poloxamer 407 and 5 mg Berberine Hydrochloride in sterile water, for 24 hours prior to harvesting and staining for flow cytometry analysis using previously published protocols (Chakraborty et al., 2021[3]). Cells were counted and 1x10^5^ cells/ml in 50 μl volume were stained for analysis. Cells from every treatment group were treated with a rat anti-mouse Fc block (CD16/CD32; 553141, 1:50 dilution; BD Horizon, Franklin Lakes, NJ) to prevent non-specific Fc binding (if any). Cells were then stained in FACS buffer (dPBS+2% HI- FCS) with PE-Cy7 Ms anti-Ms I-Ab (MHCII, clone AF6-120.1, Biolegend, catalogue no. 116419, 1:500), PerCP-Cy5.5 Ra anti-Ms Ly-6G/Ly-6C (GR1, clone RB6-8C5, BD, catalogue no. 552093, 1:200), PE-CG594 Ra anti-Ms CD40 (clone GL1, BD, catalogue no. 562847, 1:200), BV786 Ra anti-Ms CD45 (clone 30-F11, BD, catalogue no. 564225, 1:100), BUV395 Hm anti-Ms CD11c (clone HL3, BD, catalogue no. 564080, 1:50), APC Fire 750 Ra anti-Ms F4/80 (clone BM8, Biolegend, catalogue no. 123151, 1:100) and Alexa Fluor 647 Ra anti-Ms CD206 (clone C068C2, Biolegend, catalogue no. 141711, 1:200). Followed by primary antibody staining, live/dead staining was performed using zombie dye (Biolegend, catalogue no. 423101, 1:1000) except on unstained and fluorescent minus one (FMO) controls. Cells were washed and fixed with dPBS+1 % PFA and acquired using a BD Fortessa X20 at Monash FlowCore Platform.

Unstained cells were used to set voltages for Forward Scatter (FSC) and Side Scatter (SSC), followed by acquiring the single cell control using compensation beads (ABC compensation beads, Invitrogen, catalogue no. A10497). Data was acquired in BD FACS Diva and analyzed using FlowJo. The extent of activation of BMDCs was measured in terms of CD40 expression characterised by the median fluorescence intensity (MFI), whereas the extent of activation of BMDMs was measured both in terms of percentage of M1-polarized macrophages (characterized as CD45^+^CD206^-^F4/80^+^ subset of BMDMs) and in terms of CD40 MFI on both CD11c^-^MHCII^+^CD45^+^Lin^-^F4/80^+ ^and CD45^+^CD206^-^F4/80^+^ cells. 

To study the anti-fibrotic and anti-remodeling effects of BM-LCNs, we utilized a TGF-β1 induced BJ3 human dermal fibroblasts (BJ3 HDF) *in vitro* model as described before (Pinar et al., 2020[9]). BJ3 HDFs were stimulated with TGF-β1 (5 ng/ml) for 72 hours for differentiation into myofibroblasts or simultaneously treated with BM-LCNs (50 μl formulation as stated above). The antifibrotic effect was investigated *via* the detection of the levels of collagen I and α-SMA proteins using Western Blot as per pre-established protocols (Chakraborty et al., 2021[3]; Pinar et al., 2020[9]). After treatment, the cells were lysed for protein extraction using 20 μl of a cocktail of 1X RIPA lysis buffer (from 10X RIPA, Cell signalling, catalogue no. 9806) with phosphatase/protease inhibitor (Cell signalling, catalogue no. 5872) and PMSF (Cell signalling, catalogue no. 8553). Samples were incubated for 1 hour on ice followed by collecting the supernatant after centrifugation at 1500RPM for 10 min at 4 °C. The total protein content was quantified via a Bradford assay. 50 µg of total protein samples from each treatment group were loaded in Nu-PAGE gels (4-12 % Bis-Tris, 1.0mm, Mini Protein gel, catalogue no. NP0326) and analyzed by Western blotting using antibodies to collagen I (Abcam, catalogue no. ab34710, 1:1000), α-SMA (DAKO/Agilent Technologies, catalogue no. M0851, 1:1000) and equivalent loading control was confirmed using the house-keeping protein, GAPDH (Abcam, catalogue no. ab8245, 1:1000). All primary antibodies were detected using either anti-rabbit or anti-mouse HRP conjugated secondary antibodies (Cell signalling Technologies) and developed using Clarity Western ECL substrate detection system for densitometric analysis with a ChemiDoc MP Imaging System and BioRad Image-Lab v6. The relative expression of collagen I and α-SMA among groups was measured upon normalization with GAPDH and expressed relative to controls. Statistical analysis was performed using GraphPad PRISM version 9.4. Two-way ANOVA test was used to compare the different experimental groups, followed by Tukey's multiple comparisons test for pairwise comparisons.

We observed that LPS stimulation significantly increased the expression of the activation marker CD40 on BMDCs (Supplementary Figure 1A, B; p<0.001). However, when treated with BM-LCNs, the upregulation of CD40 on these DCs was significantly reduced compared to the LPS-stimulated group (Supplementary Figure 1A, B; p<0.01). This result suggested that BM-LCNs effectively inhibited the activation of dendritic cells induced by LPS.

Next, we examined the impact of BM-LCNs on modulating CD40 expression in BMDMs. LPS stimulation led to a significant upregulation of CD40 expression in BMDMs (Supplementary Figure 1C, D; p<0.001). However, when treated with BM-LCNs, the expression of CD40 was significantly reduced back to baseline levels (Supplementary Figure 1C, D; p < 0.001). This finding indicated that Berberine nanoparticles effectively counteracted the LPS-induced increase in CD40 expression in macrophages thereby acting as an immunomodulator.

To assess the impact of BM-LCNs on macrophage polarization, we examined the CD45^+^CD206^-^F4/80^+^ subset of BMDMs, which represented M1-skewed macrophages. Upon LPS stimulation, we observed a significant 6-fold increase in the proportion of M1 macrophages (Supplementary Figure 1E, F; p<0.05). However, treatment with BM-LCNs induced a strong trend towards a reduction in M1 macrophage infiltration (p = 0.0539), although this did not result in a significant reduction (Supplementary Figure 1E, F). Despite not showing a significant reduction in the proportion of M1 macrophages, BM-LCNs had a notable effect on the activation marker CD40 within this subset of BMDMs. Treatment with BM-LCNs led to a significant reduction in CD40 expression in M1 macrophages compared to the LPS-stimulated group (Supplementary Figure 1G, H; p<0.001). These results demonstrated that BM-LCNs induced skewing of M1 macrophages to naïve macrophages by alleviating pro-inflammatory mediators.

Finally, we investigated the anti-fibrotic and anti-remodeling role of BM-LCNs on TGF-β1-induced BJ3 HDFs. TGF-b1 stimulated a significant increase in α-SMA-associated myofibroblast differentiation by ~2.5 fold (p < 0.05; Supplementary Figure 2A, B) and collagen I expression by ~3.5 fold (p< 0.001; Supplementary Figure 2A, C) after 72 hours. BM-LCNs induced a strong trend towards preventing the TGF-β1-induced increase in α-SMA expression (p=0.068; Supplementary Figure 2A, B) and significantly prevented the TGF-β1-induced increase in collagen I expression after 72 hours of treatment (Supplementary Figure 2A, C; p<0.001). These results demonstrated the anti-fibrotic and plausible anti-remodeling effects of BM-LCNs.

These findings add to our previous studies that have demonstrated the therapeutic potential of berberine-LCNs to inhibit β-catenin activity, a crucial stimulator of epithelial-to-mesenchymal transition (Malyla et al., 2023[6]), and anti-inflammatory activity of berberine-LCNs against LPS induced RAW264.7, an immortalized murine macrophage cell line (Alnuqaydan et al., 2022[1]). In the current study, the anti-inflammatory activity of berberine in BMDMs, and its anti-fibrotic and anti-remodeling effects in HDFs was demonstrated. As expected, we observed a good correlation between the anti-inflammatory activity of berberine in immortalized cells (RAW264.7) and BMDMs in the current study in response to the same stimulant (LPS). Taken together, the promising anti-inflammatory and anti-fibrotic activity of berberine-LCNs should be further validated in pre-clinical animal models followed by clinical trials for translational research from bench side to bedside.

## Notes

Amlan Chakraborty and Keshav Raj Paudel contributed equally as first author.

Amlan Chakraborty and Kamal Dua (Discipline of Pharmacy, Graduate School of Health, University of Technology Sydney, Ultimo, NSW 2007, Australia; E-mail: Kamal.Dua@uts.edu.au) contributed equally as corresponding author.

## Conflict of interest

The authors declare that they have no conflict of interest.

## Supplementary Material

Supplementary information
