# Supplementary material for: Anti-inflammatory and anti-fibrotic effects of berberine-loaded liquid crystalline nanoparticles
Source: EXCLI J. 2023 Oct 26;22:1104–8. doi: 10.17179/excli2023-6467 (PMC10711197; doi:10.17179/excli2023-6467)
Supplement: Supplementary information [file EXCLI-22-1104-s-001.pdf]

EXCLI Journal 2023;22:1104-1108 – ISSN 1611-2156
Received: August 13, 2023, accepted: October 18, 2023, published: October 26, 2023

Supplementary information to:

Letter to the editor:

ANTI-INFLAMMATORY AND ANTI-FIBROTIC EFFECTS OF BERBERINE-LOADED LIQUID CRYSTALLINE NANOPARTICLES

Amlan Chakraborty[1,2,#,*], Keshav Raj Paudel[3,#], Chao Wang[2], Gabriele De Rubis[4,5], Dinesh Kumar Chellappan[6], Philip Michael Hansbro[3], Chrishan S. Samuel[2], Kamal Dua[4,5,*]

1 Faculty of Biology, Medicine and Health, The University of Manchester, Oxford Road, Manchester M13 9PL, U.K.
2 Cardiovascular Disease Program, Biomedicine Discovery Institute and Department of Pharmacology, Monash University, Clayton, VIC 3800, Australia
3 Centre for Inflammation, Centenary Institute & University of Technology Sydney, School of Life Sciences, Faculty of Science, Sydney, New South Wales 2050 & 2007, Australia
4 Discipline of Pharmacy, Graduate School of Health, University of Technology Sydney, Ultimo, NSW 2007, Australia
5 Faculty of Health, Australian Research Centre in Complementary and Integrative Medicine, University of Technology Sydney, Ultimo, NSW 2007, Australia
6 Department of Life Sciences, School of Pharmacy, International Medical University (IMU), Bukit Jalil, Kuala Lumpur, 57000, Malaysia

# Equal contribution
* **Corresponding authors:** Dr. Amlan Chakraborty, Faculty of Biology, Medicine and Health, The University of Manchester, Oxford Road, Manchester M13 9PL, U.K. E-mail amlan.chakraborty@manchester.ac.uk
Dr. Kamal Dua, Discipline of Pharmacy, Graduate School of Health, University of Technology Sydney, Ultimo, NSW 2007, Australia. E-mail: Kamal.Dua@uts.edu.au

https://dx.doi.org/10.17179/excli2023-6467

This is an Open Access article distributed under the terms of the Creative Commons Attribution License (http://creativecommons.org/licenses/by/4.0/).

EXCLI Journal 2023;22:1104-1108 – ISSN 1611-2156
Received: August 13, 2023, accepted: October 18, 2023, published: October 26, 2023

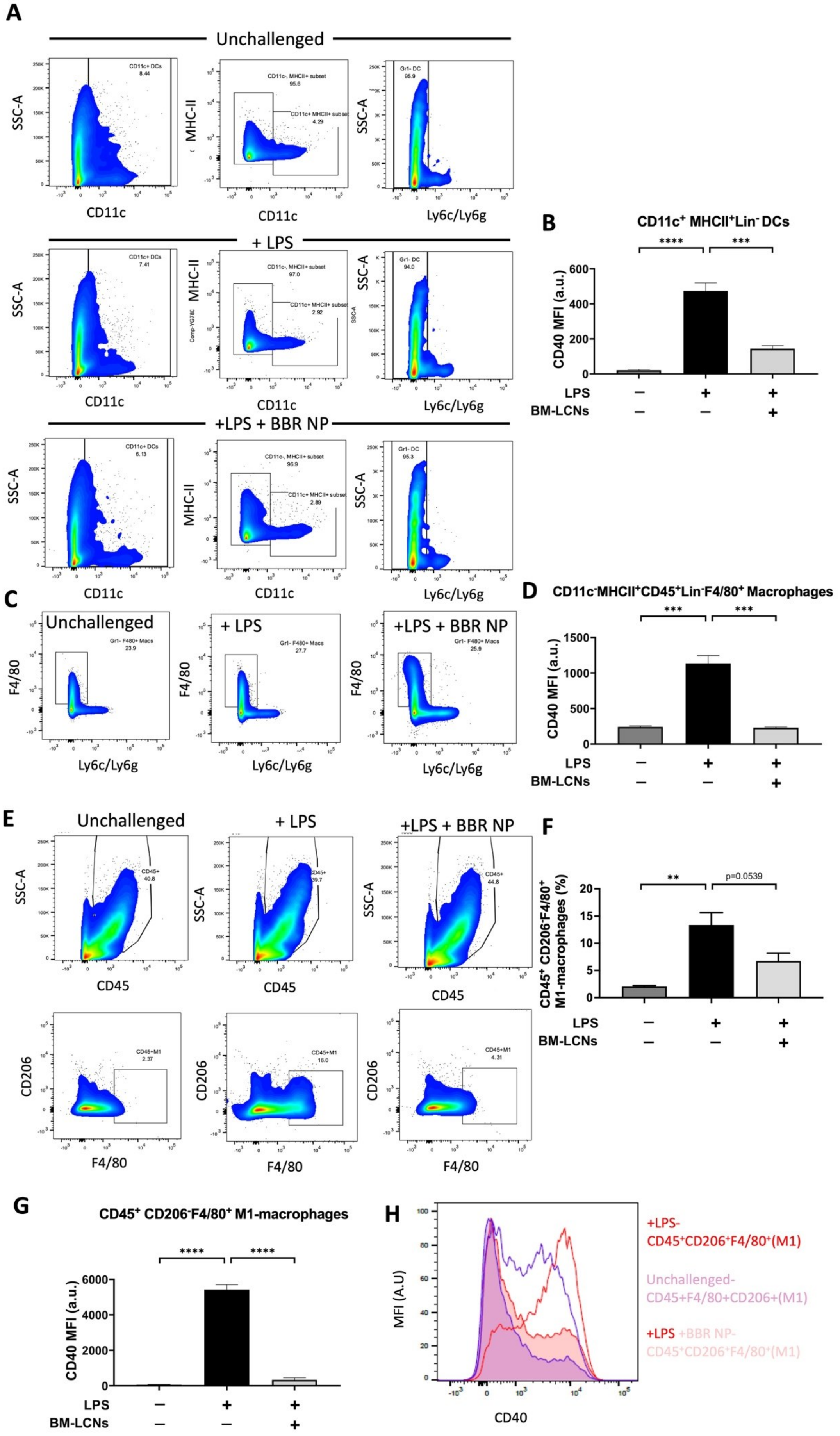

EXCLI Journal 2023;22:1104-1108 – ISSN 1611-2156
Received: August 13, 2023, accepted: October 18, 2023, published: October 26, 2023

Figure 1: BM-LCNs counteract LPS-induced Dendritic cell activation and macrophage. (A, B): BMDCs were treated with 100 ng/ml LPS for 24 hours and then exposed to BM-LCNs (100 ml formulating with 1 ml formulation containing 0.20 g Monoolein, 0.02 g Poloxamer 407, 5.00 mg Berberine Hydrochloride in sterile water) for 24 h. **(A):** representative dot plots showing the gating strategy employed for the analysis of CD40 expression on CD11c^{+}MHCII^{+}Lin^{-} BDMCs; **(B):** analysis of CD40 MFI on CD11c^{+}MHCII^{+}Lin^{-} BDMCs. **(C, D):** BMDMs were treated with 100 ng/ml LPS for 24 hours and then exposed to BM-LCNs (same concentration as stated before) for 24 hours. **(C):** representative dot plots showing the gating strategy employed for the analysis of CD40 expression on CD11c^{-}MHCII^{+}CD45^{+}Lin^{-}F4/80^{+} BMDMs; **(D):** analysis of CD40 MFI on CD11c^{-}MHCII^{+}CD45^{+}Lin^{-}F4/80^{+} BMDMs. **(E-H):** BMDMs were treated with 100 ng/ml LPS for 24 hours and then exposed to BM-LCNs for 24 hours. **(E):** representative dot plots showing the gating strategy employed for the analysis of the percentage of M1-skewed macrophages, defined as the CD45^{+}CD206^{-}F4/80^{+} subset of BMDMs; **(F):** analysis of the percentage of CD45^{+}CD206^{-}F4/80^{+} M1-skewed macrophages; **(G):** analysis of CD40 MFI on CD45^{+}CD206^{-}F4/80^{+} M1-skewed macrophages; **(H):** representative histogram showing the relative CD40 MFI in CD45^{+}CD206^{-}F4/80^{+} M1-skewed macrophages. Statistics: One-Way ANOVA (n = 3). **: p<0.01; ***: p<0.001; ****p<0.0001.

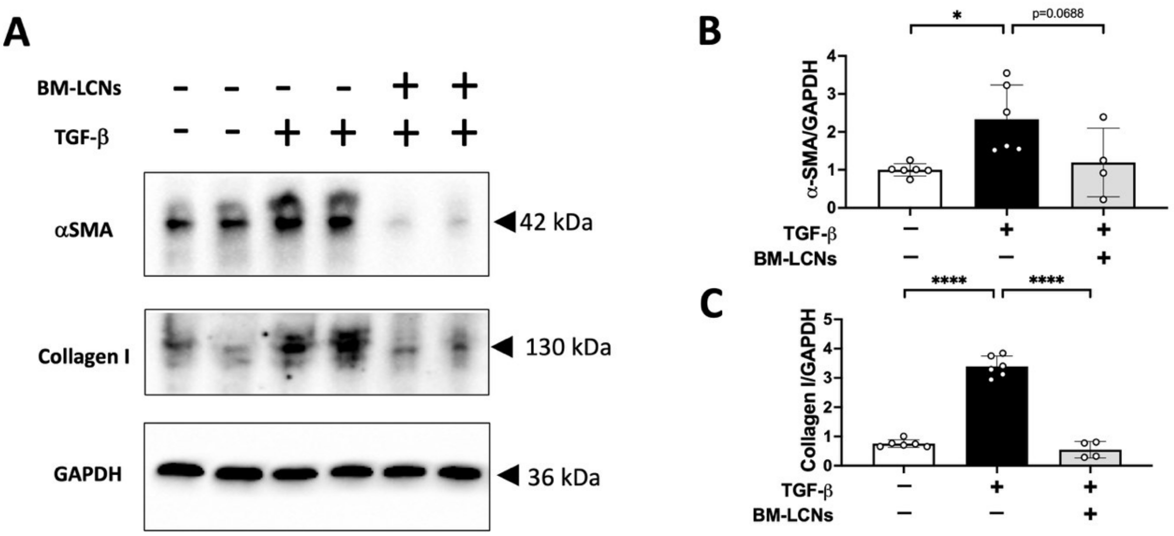

Supplementary Figure 2: BM-LCNs counteract TGF-β-induced expression of α-SMA and Collagen I in BJ3 Human Dermal Fibroblasts. BJ3 HDFs were treated with 5 ng/ml TGF-β1 for 72 hours and exposed to BM-LCNs (100 ml formulating with 1 ml formulation containing 0.20 g Monoolein, 0.02 g Poloxamer 407, 5.00 mg Berberine Hydrochloride in sterile water). (**A**): representative western blots showing the effect of treatments on the expression of α-SMA, Collagen I, and GAPDH. (**B**): analysis of the relative expression of α-SMA; (**C**): analysis of the relative expression of Collagen I. Values in (**B**) and (**C**) are normalized by GAPDH expression. Statistics: One-Way ANOVA (n = 4-6). **: p<0.01; ***: p<0.001; ****p<0.0001.
